# Convergence to consensus in heterogeneous groups and the emergence of informal leadership

**DOI:** 10.1038/srep29704

**Published:** 2016-07-14

**Authors:** Sergey Gavrilets, Jeremy Auerbach, Mark van Vugt

**Affiliations:** 1 Department of Ecology and Evolutionary Biology, University of Tennessee, Knoxville, TN 37996, USA; 2 Department of Mathematics, University of Tennessee, Knoxville, TN 37996, USA; 3 National Institute for Mathematical and Biological Synthesis, University of Tennessee, Knoxville, TN 37996, USA; 4 Department of Social and Organizational Psychology, VU University of Amsterdam, Amsterdam, Netherlands

## Abstract

When group cohesion is essential, groups must have efficient strategies in place for consensus decision-making. Recent theoretical work suggests that shared decision-making is often the most efficient way for dealing with both information uncertainty and individual variation in preferences. However, some animal and most human groups make collective decisions through particular individuals, leaders, that have a disproportionate influence on group decision-making. To address this discrepancy between theory and data, we study a simple, but general, model that explicitly focuses on the dynamics of consensus building in groups composed by individuals who are heterogeneous in preferences, certain personality traits (agreeability and persuasiveness), reputation, and social networks. We show that within-group heterogeneity can significantly delay democratic consensus building as well as give rise to the emergence of informal leaders, i.e. individuals with a disproportionately large impact on group decisions. Our results thus imply strong benefits of leadership particularly when groups experience time pressure and significant conflict of interest between members (due to various between-individual differences). Overall, our models shed light on why leadership and decision-making hierarchies are widespread, especially in human groups.

All group-living animals, including humans, regularly need to coordinate their activities with group-mates. For example, animal groups must decide on where and when to collectively eat, drink, rest, and move^1^,^2^. Such decisions can have important consequences for group cohesion and the fitness of individual members. Thus, group living requires mechanisms to engage in effective group decision-making. An inherent feature of both animal and human groups is their heterogeneity. Some group members are perhaps better informed than others, for instance, about the location of a foraging site, and there may be conflicting preferences among group members on which site to go to. Understanding how groups aggregate information — while accounting for variation in individual preferences and personalities — represents a big theoretical and applied challenge across both the biological and social sciences.

Recent theoretical work in evolutionary biology suggests that shared, consensus-based decision-making is often the most efficient way for dealing with both information uncertainty and between-individual variation in preferences^3^. This conclusion is in line with theoretical models on swarm intelligence, “wisdom of crowds”, quorum seeking, and voting in elections^4^. However, some animal groups make collective decisions through particular individuals – or leaders – that have a disproportionate influence on group decision-making. For instance, in elephants, dolphins, killer whales, and ravens the most knowledgeable group member often dictates group movement^5^,^6^. Anthropological research suggests that virtually all groups and societies have leaders guiding collective decision-making in place, although they may be situational^7^,^8^. In modern human societies, decision-making hierarchies are ubiquitous: nations have politicians, businesses have executives, and classrooms have teachers. In fields such as economics, social psychology, organizational and political science, researchers have shown the benefits of decision-making hierarchies^9^.

So why are decision-making hierarchies common in animals and ubiquitous in humans^10^ when theoretical models (e.g., swarm intelligence, wisdom of crowds) favor shared, consensus-based decision-making procedures? The discrepancy between theory and practice was not lost in Conradt’s^3^ review that noted that “an interesting question is why so many empirical studies report unshared and dictatorial decision-making, while the models predominantly predict shared decisions. Obviously, the models are missing some aspects of the problem” [p.238].

There are at least three important factors that are absent or underdeveloped in existing models and that can influence the distribution of leadership in consensus building. First, variation in dominance, personality, or experience contribute to the formation of stable hierarchies resulting in some individuals being more “influential” than others^6^. For example, in fish, differences in temperament give rise to a relatively stable social structure in which leaders move first thus coordinating movement^11^. In non-human primates, physically dominant individuals dictate group movement because they are more autonomous and coersive^12^,^13^. Individuals who are more stubborn and less agreeable – and thus less willing to shift their position – are more likely to emerge as leaders^14^,^15^,^16^. In humans, behavioral genetics research shows that such personality differences have a substantial heritable component^17^,^18^,^19^.

A second contributing factor to the emergence of leadership and hierarchy is time constraints. Reaching consensus via a democratic process takes time which might be quite costly for groups facing certain challenges. Human research shows that as groups face time pressure they move from a shared to an unshared, hierarchical decision-making structure^20^. For example, in the Robbers Cave experiment, a classic psychological study on intergroup behavior during a summer camp, Sherif *et al*.^21^ showed that when the two groups became aware of each other’s presence they each appointed team captains. This is echoed by anthropological evidence that egalitarian hunter-gatherer groups revert to a more hierarchical structure when swift action is needed to deal, for instance, with aggressive outgroups^22^.

A third, related factor conducive to the emergence of leadership and hierarchy is group size. In larger human groups, social inequalities tend to be greater, which results in differences in power and influence between members^23^,^24^. In addition, individuals in larger groups often have dispersed social networks. Establishing consensus via shared decision-making will be harder in such groups as individuals cannot interact with everyone else at the same time. Thus, deferring to a leader is an appropriate solution in large, dispersed groups, provided that there are mechanisms in place that help groups decide which individuals to follow. Reputation-building may be a key mechanism for leadership selection^25^.

Our paper addresses the theoretical gap identified above using a simple, but general, model that explicitly focuses on the time it takes to reach a consensus in groups composed by individuals who are heterogeneous in personality traits, reputation, and social networks. Theoretical work studying the time to consensus actually has quite a long history in mathematics and physics, where it comes under the rubric of the voter model^26^,^27^,^28^. Although this important work has been widely used across the biological and social sciences, it has been ignored in the biological literature on group decision-making. In its most basic version, individuals have opinions which can be specified by integers. Individuals interact stochastically in dyads and upon an interaction one individual of the pair accepts the opinion of their partner. Under certain conditions, the whole group converges on a single opinion in finite time. Mathematically, the voter model is quite similar to the model of random genetic drift in population genetics^29^, with the time to consensus in the former corresponding to the time to genetic fixation in the latter. Literature on the voter model in general is quite extensive^30^. While most of the earlier research focused on symmetric models with identical individuals, more recent work has allowed for heterogeneity of individuals with respect to their personality traits and social networks^26^,^27^,^28^,^31^,^32^,^33^.

This work, however, has application to only some types of individual and group behaviors. Typically in voter models, (1) there is only a small number of discrete opinions (usually just two), (2) individuals accept the opinion of their partner completely, (3) consensus is defined as complete uniformity of opinions in the group, and (4) the focus is on infinitely large groups. In contrast, in real-life decisions such as group movement, preferences can vary continuously and individuals can modify their preferences in the direction of their partner but are not always willing to accept their opinion immediately^13^,^34^. Because complete convergence of opinions would here take very long time, it makes more sense to define consensus as a state in which the differences in opinions have been significantly reduced (e.g., below a certain threshold), rather than eliminated completely. The dynamics of the opinion convergence versus that of complete disappearance of variation in opinions (“fixation”) proceed on different time scales^26^,^27^,^28^,^31^. It takes much more time to entirely eliminate variation in opinion than to reduce it below some threshold. Most theoretical work has focused on the time to “fixation” (but see ref. 35). Finally, experiments show that even very small groups (i.e fewer than 6–8 individuals) often switch from consensus to leadership^36^ so that the large group size limit usually used in the voter model may not be justified. These limitations of existing mathematical work imply that additional studies of biologically inspired models are needed.

Our models below will explicitly focus on the dynamics of consensus building in relatively small, heterogeneous groups while largely ignoring the question of decision accuracy and the individual costs (but see the end of the main section). Our approach is thus complementary to earlier approaches (mentioned above) that focus on either decision accuracy or individual costs but ignore the time component and/or do not consider explicitly how the actually process of convergence to a consensus decision occurs. Although jointly accounting for time, precision and costs in a single framework is clearly most desirable, ignoring the latter two is justifiable in certain situations. For example, in small-scale acephalous societies individuals may have different preferences over the timing of a camp move or initiation of a hunt, but the benefit of acting in a coordinated fashion clearly outweighs other considerations. In emergency situations such as wars or natural disasters, groups often face a trade-off between decision accuracy and decision speed. In such situations it could be highly beneficial to speed up the decision process and to ignore that some individuals have different preferences over the course of action.

## Models and Results

### Consensus building

We consider a group of *n* individuals who differ with respect to their preferences, specified by scalar numbers *x*_*i*_ (*i* = 1, …, *n* and *x*_*i*_ in (0, 1)). The individuals are motivated to reach a consensus and are willing to change their preferences. That is, group consensus and cohesion is of higher priority to everyone than acting according to initial individual preferences. We model the consensus building as a sequence of “events”. At each event, a particular “listener”, say, individual *i*, listens to the argument of a particular “speaker”, say, individual *j*, and then modifies their preference in the direction of that of the speaker. Specifically, after the event the preference of the listener becomes


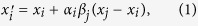


where parameter *α*_*i*_ measures the listener’s willingness to change their opinion and parameter *β*_*j*_ the speaker’s ability to change the listener’s opinion. We will refer to *α*_*i*_ as “agreeability” and to *β*_*j*_ as “persuasiveness”. The larger *α*_*i*_ and *β*_*j*_ are, the more significant change in the listener’s preference after the event. We will treat *α*_*i*_ and *β*_*j*_ as individual-specific constant traits and will allow for variation between individuals in these traits. It is natural to assume that all *α*_*i*_ and *β*_*j*_ are between 0 and 1. Note that a typical setup in the voter model implies that *α*_*i*_*β*_*j*_ = 1 so that the listener adopts the preference of the speaker. In a model studied in ref. 35, both members of the dyad change their preferences simultaneously to choose the middle preference, so that *α*_*i*_*β*_*j*_ = 0.5. Our assumptions imply that the group does not have a formal mechanism of (or an institution for) achieving a joint decision (such as democratic voting with votes being counted and/or somehow averaged). Rather a consensus decision emerges by informal interactions between individuals as happens in many foraging groups^37^,^38^.

**Figure 1:**
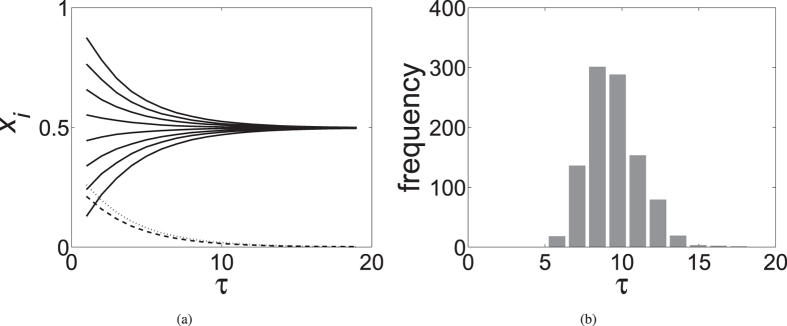
Reaching consensus in a group of *n* = 8 individuals each with *α*_*i*_ = *β*_*i*_ = 0.25. The initial preferences are spaced uniformly between 0 and 1. (**a**) The average dynamics of individual preferences (eight solid lines) and standard deviation and average absolute distance to the consensus value x^*^ = 0.5 (two dashed lines). (**b**) The distribution of the time to consensus *τ* (defined as the time when the average absolute distance to x^*^ drops below 1/10th of the initial value). 1000 runs, *μ* = 1.

We will assume that at each event the listener is chosen randomly out of *n* group members. The speaker is then chosen out of the *n* − 1 remaining group members uniformly randomly or with probabilities dependent on some individual characteristics (such as reputation or social relatedness to the speaker; see below). After a sufficiently large number of events, individual preferences *x*_*i*_ will converge to a consensus value, say x^*^ (see Fig. 1). We are interested in approximating x^*^ as well as in estimating the characteristic time to convergence *τ*.

We assume that on average each individual plays the role of listener *μ* times during the unit of time. (For example, if group members are spatially dispersed, the encounter rate *μ* can be low.) Our unit of time then corresponds to *n*/*μ* events. In the Supplementary Material (SM) we show that the preferences *x*_*i*_ will converge to x^*^ exponentially in time. Therefore it makes sense to measure the characteristic time to convergence *τ* using the mean lifetime of an exponential decay process which in turn can be approximated by the maximum eigenvalue of a corresponding matrix. It also makes sense to measure within-group variation in preferences using the mean absolute deviation rather than standard deviation. The time for the variation in individual preferences to reduce to, say, one tenth of its initial value will then be ln(10) × *τ*. Note that by definition *τ* does not depend on the initial variation in preferences. Increasing the latter by a factor of *k*, will result in increasing the time to reach a specific level of variation in preferences by a factor ln(*k*).

### Random dyads

Let the speaker-listener pairs be chosen completely randomly. We show in the SM that in this case the consensus value x^*^ can be approximated as


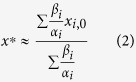


(ref. 27). This implies that the initial preferences *x*_*i*,0_ of individuals with the highest ratio of persuasiveness *β*_*i*_ to agreeability *α*_*i*_ will have the highest weights in the consensus value x^*^. We can say thus that the most influential individuals will be those with the largest *β*_*i*_/*α*_*i*_ ratio.

To evaluate the characteristic time to convergence *τ*, assume first that individuals do not differ in agreeability (i.e that *α*_*i*_ = *α* for all *i*). In this case, *τ* can be approximated as


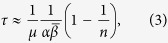


where 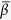 is the average persuasiveness in the group (see the SM). That is, the smaller individual agreeability *α* and the smaller average persuasiveness 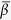, the longer it takes to build a consensus. This makes intuitive sense. Increasing the group size *n* increases *τ* but only weakly (less than by a factor 2). Although larger groups require more dyadic interactions to build a consensus, more “events” occur within a unit of time which reduces the overall effects of the group size.

When individuals differ in their agreeability (i.e. *α*_*i*_ are different), the characteristic time to convergence *τ* can be approximated as


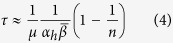


where *α*_*h*_ is the mean harmonic agreeability in the group (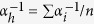). Because individuals with the smallest *α*_*i*_ makes the largest contribution to *α*_*h*_, with variation in agreeability, the time scale to convergence is largely controlled by the most “stubborn” group members. Again, this makes intuitive sense. Larger groups are more likely to harbor individuals with more extreme personality characteristics, and we expect *α*_*h*_ to decrease with *n* to a limit. As a result, the time to convergence will grow with the group size. Therefore, in this case *τ* will grow much faster with *n* than in the case of equal *α*_*i*_. We conclude that the group size effect becomes sufficiently important only in groups that have some individuals with very low agreeability. Our numerical simulations show that approximations (3) and (4) somewhat underestimate *τ*. As noted by a reviewer, while the expectations of *x*_*i*_s converge to x^*^, the actual distribution of *x*_*i*_’s in any particular realization depends on the parameters of the model: it may be narrow or broad, and may even be bimodal (e.g., if there are more than two very stubborn individuals).

**Figure 2:**
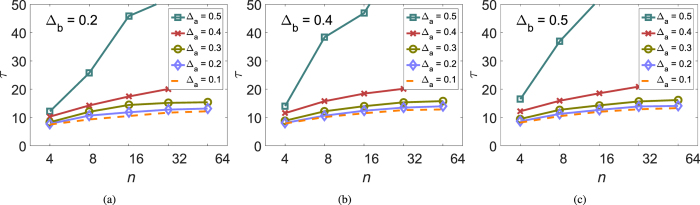
The dependence of the time to consensus *τ* from simulations on group size *n* and variation in agreeability *α*_*i*_ and persuasiveness *β*_*i*_. Individual values of *α*_*i*_ and *β*_*i*_ were chosen independently with a uniform probability from intervals of length Δ_*a*_ and Δ_*b*_ centered on 0.5. (**a**) Δ_*b*_ = 0.2. (**b**) Δ_*b*_ = 0.4. (**c**) Δ_*b*_ = 0.5. 5,000 simulations were run for each parameter combination with random formation of dyads; initial preferences were drawn from a uniform distribution on the interval (0, 1).

Figure 2 illustrates our results numerically. In our simulations, individual values of *α*_*i*_ and *β*_*i*_ were chosen randomly and independently with a uniform probability from intervals centered on 0.5 with width Δ_*a*_ and Δ_*b*_, respectively. That is, parameters Δ_*a*_ and Δ_*b*_ characterize the maximum spread of *α*_*i*_ and *β*_*i*_. As predicted, the graphs grow approximately linearly only with the largest value of Δ_*a*_ while for smaller Δ_*a*_ they saturate. Comparing the graphs corresponding to different values of Δ_*b*_ shows that the extent of variation in persuasiveness *β*_*i*_ has not much effect on the characteristic time to consensus *τ*.

### Effects of reputation

Instead of assuming random encounters of speakers and listeners, now assume that speakers are chosen according to their reputation or status. Let each individual have a normalized reputation *ρ*_*i*_ (so that ∑*ρ*_*i*_ = 1). Then given that individual *i* is the listener, let the probability that individual *j* is chosen as the speaker be *ρ*_*j*_/∑_*j*≠*i*_*ρ*_*j*_. Note that if all individuals have the same reputation (*ρ*_*i*_ = 1/*n*), the last expression simplifies, to 1/(*n* − 1) as in our basic model.

Assume first that individuals have equal agreeability (*α*_*i*_ = *α*) and persuasiveness (*β*_*i*_ = *β*), but differ in reputation *ρ*_*i*_. Then the consensus value is approximated as


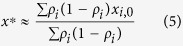


(ref. 27). The largest influence on x^*^ will be an individual with the largest value of *ρ*_*i*_(1 − *ρ*_*i*_), which will be the individual with the largest reputation *ρ*_*i*_ (since ∑ *ρ*_*i*_ = 1).

Within the realm of our approximation, *τ* does not depend on the distribution of *ρ*_*i*_ if there is no variation in *α*_*i*_ and *β*_*i*_. Numerical simulations show that although this is not true in general, the dependence is relatively weak, see Figure S7 in the SM. In numerical simulations, reputation values *ρ*_*i*_ were chosen randomly and independently from the symmetric Dirichlet distribution with concentration parameter *γ*. (With *γ* = ∞, all individuals have equal reputation *ρ*_*i*_ = 1/*n*; with *γ* = 1, the distribution of *ρ*_*i*_ is uniform; with small *γ*, most individuals have very low reputation while one or maybe two individuals have large reputation). Figure S9 shows an unexpected crossover effect: groups with small variation in reputations *ρ*_*i*_ are more sensitive to the group size than groups with high variation in reputation. Intuitively, in the latter case, consensus building is largely mediated by the interactions between an individual with the highest reputation and the rest of the group who have low reputation. The exact number of low-reputation individuals is not that important because their opinions change in a similar fashion. We conclude that in large groups the presence of one or few highly reputable individuals (who can be viewed as informal leaders) helps to achieve the consensus quicker but the overall effect is small.

### General case

Approximations and results for the case when individuals differ with respect to agreeability *α*_*i*_, persuasiveness *β*_*i*_, and reputation *ρ*_*i*_ are given in the SM. The largest influence on the consensus values is by the individual with the largest value of (*β*_*i*_/*α*_*i*_)*ρ*_*i*_. The time to convergence is now controlled by the individual characterized by a combination of small agreeability and high reputation resulting in the minimum value of *α*_*i*_/(1 − *ρ*_*i*_).

### Multiple listeners

So far we have assumed that there was always a single listener per speaker. With multiple listeners per speaker one expects that consensus will be achieved faster. This is indeed observed in numerical simulations (see the SM). Our results show with ℓ listeners the time to consensus reduces approximately by a factor 1/ℓ, as one may expect.

### Social networks

So far we have assumed that each individual can directly interact with any other group member. Now we assume that each individual interacts only with a limited number of his/her specific social partners. Let as before at each event a single listener be chosen randomly. But now we posit that for each listener, the speaker is chosen randomly and uniformly from the set of social partners.

**Figure 3:**
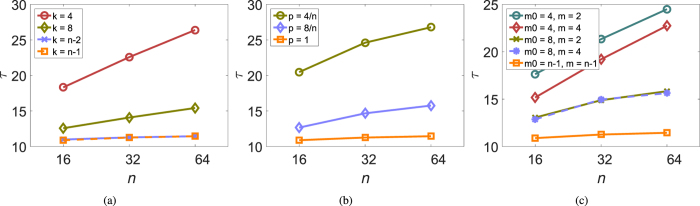
The average time to reach consensus for 3 different types of random networks. (**a**) Each group member has the same number of connections *k*. (**b**) Erdös-Rényi networks with different probabilities of connectedness *p*. (**c**) Preferential attachment networks with varying initial complete networks of size *m*_0_ and *m* connections for each additional group member. No variation in personality traits (*α*_*i*_ = *β*_*i*_ = 0.5 for all *n* individuals).

We investigated three different types of social networks assuming there is no variation in personality traits (Fig. 3; see the SM for more analytical and numerical results). Figure 3a shows the average time to reach consensus for random networks where each group member has the same number *k* of connections (i.e. social partners). Reducing the number of connections *k* increases the time to reach consensus. Figure 3b corresponds to the case of Erdös-Rényi networks^39^ where each pair of individuals are connected with probability *p*. Results are similar to the fixed connection model: decreasing *p* will decrease the number of connections in the group and increase the time to consensus. Figure 3c corresponds to preferential attachment networks^40^. In building these networks we followed ref. 39 by starting with completely connected networks of *m*_0_ individuals and then choosing exactly *m* connections for each additional group member (*m* < *m*_0_). Reducing the size of the initial complete network *m*_0_ and therefore reducing the number of highly connected individuals dramatically increases the time to reach consensus. This effect is similar to that found when reducing the number of connections in the other models. We conclude that reducing the number of social partners can significantly delay the consensus. As expected, individuals with the largest number of connections have disproportionately large impacts on the consensus value with the overall effect being comparable to that of reputation (see Figure S14 in the SM).

### Reputation dynamics

Our results above show that reaching a consensus can take significant time especially if there is enough variation in agreeableness such there are a number of highly stubborn individuals in the group, which would be more likely as group size increased. If this results in a large cost for individuals and the group, then delegating the decision to a single individual (a leader) may be a better alternative than attempting to reach a consensus. But who should be a leader? One possibility is that this should be an individual who has attained the highest reputation for making a right decision.

Assume that before the group takes a certain action, each individual expresses the best action in their opinion. Assume that after the action the group knows what the best solution actually was. Then each individual can be assigned a value *c*, which we will refer to as “capability”, measuring their ability to make a good decision. We expect that individuals with higher than average capability will increase their reputation *ρ* while those with lower than average capability will decrease it. This can be captured by a simple model describing how individual reputation changes after each group action:


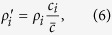


where 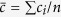 is the average capability in the group. An advantage of our approach is that the above equation for *ρ*_*i*_′ is identical to that of the one-locus multi-allele population genetic model of natural selection whose dynamics are completely understood once the “capabilities” *c*_*i*_ are specified^29^,^41^.

**Figure 4:**
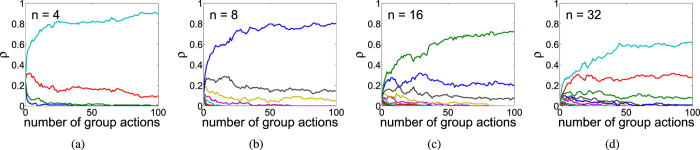
Reputation dynamics for different group sizes (averaged over 5,000 runs). (**a**) *n* = 4. (**b**) *n* = 8. (**c**) *n* = 16. (**d**) *n* = 32. Initial reputations were equal (*ρ*_*i*_(0) = 1/*n*) and capabilities *c*_*i*_ were drawn from a beta distribution with parameters (2, 5).

Let individuals be ordered so that *c*_1_ > *c*_2_ > . . . > *c*_*n*_, so that individual #1 has the highest capability, individual #2 the second highest, and so on. Then from population genetic models, we know that a single individual with the highest reputation *ρ*_*i*_ ≈ 1 (an “informal leader”) will emerge on the time scale order 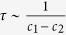, with time being measured by the number of group actions. Figure 4 illustrates the dynamics of reputations assuming that the individual capabilities *c*_*i*_ stay the same for each action. Note that the distribution of reputations that emerges corresponds to low values of *γ* (e.g. *γ* = 0.1) in the previous section. In this case, the time to reach consensus takes longer for small groups than for groups with a more uniform distribution of reputations, yet when group size gets larger the time to reach consensus is quicker (see Figure S9).

In what kind of decision-making problems do group leaders play a role? Groups generally face two kinds of cooperative challenges: (a) information tasks (e.g., where the group moves next) and (b) distribution tasks (e.g., how group members share the meat). Arguably these problems require different kinds of leaders. Information problems require the selection of leaders with knowledge and experience, whereas distribution challenges require fair and impartial leaders^23^. Yet these may not be the same individuals. Studies of leadership in hunter-gatherer societies show that leadership roles are often distributed across various individuals^8^. For instance, the Shoshone Indians have different leaders for hunting small game versus large game^22^. The Cheyenne had different leaders for war and peace: war leaders are younger, more aggressive, physically stronger, and of lower social class, whereas peace leaders are older, physically less formidable, and from more prestigious families^42^. Therefore there are at least two interesting possibilities for specifying capabilities *c*_*i*_.

### The leader is knowing

Assume that the optimum action for the group *x*_*opt*_ is exogenously specified (“information problems”). Without any loss of generality we can set *x*_*opt*_ to 0. Assume that each individual estimates *x*_*opt*_ with an error which has zero mean and variance 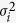. Then the individual’s “capability” can be defined as


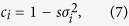


where *s* is a constant scaling parameter.

### The leader is fair

Assume that the optimum action *x*_*opt*_ is the one that is viewed as “fair” by group members (“distribution problems”). A natural candidate for a fair action is the one that minimizes the sum of squared deviations from initial individual preferences ∑_*i*_(*x*_*i*,0_ − *x*_*opt*_)^2^. This is the average, *x*_*opt*_ = ∑_*i*_*x*_*i*,0_/*n*, where *x*_*i*,0_ is the initial preference of individual *i*^43^. Assume that each individual attempts to estimate the fair action, i.e. *x*_*opt*_, by interacting with a random subset of *n*′ individuals modifying their preference according to our model (eq. 1). Let 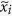 be the individual preference after this sampling process which the individual offers as his opinion on the best group action. After the action is taken, group members know what the optimum action *x*_*opt*_ actually was. In this case, individual capability can be defined as


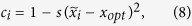


where *s* is a constant scaling parameter.

**Figure 5:**
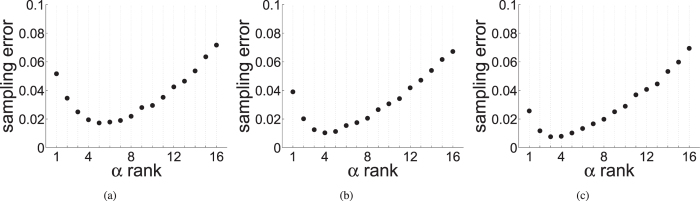
Average sampling errors, 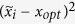, for individuals attempting to estimate the average initial preference *x*_*opt*_ by randomly sampling *n*′ group mates in a group of size *n* = 16. Individuals are ranked by their relative agreeability within the group from the lowest on the left to the highest on the right. Individual agreeability was chosen with a uniform probability from the interval (0, 1) and persuasiveness was fixed at 1. (**a**) *n*′ = 4, (**b**) *n*′ = 8, and (**c**) *n*′ = 16. 5,000 runs for each parameter combination with random formation of dyads and initial preferences drawn from a uniform distribution on the interval (0, 1).

In Fig. 5 we illustrate how individual agreeability *α* affects the individual’s ability to estimate *x*_*opt*_ using the sampling procedure outlined above. This Figure shows that there is an optimal agreeableness when attempting to estimate the average preference. This optimum value is relatively small. Intuitively, for very agreeable individuals their estimate of *x*_*opt*_ will be close to that of the last individual(s) they interacted with rather than the sampling mean. For very stubborn individuals their estimate of *x*_*opt*_ will remain close to their own initial preference *x*_*i*,0_. That is, for distribution problems, where minimizing individual costs within the group is a priority, the most effective individual is highly stubborn (but not the most stubborn). This result corresponds with observations of contemporary leadership traits^14^,^15^,^16^.

## Discussion

Earlier evolutionary models of group decision-making^2^,^43^ stressed that shared decision-making procedures often work best to achieve consensus, because they minimize the costs of individual differences in preferences. This may be true in theory, yet in reality unshared, hierarchical decision-making procedures are the norm in various species, including humans^44^. Our paper investigates joint effects of three factors that may favor hierarchical decision-making: within-group heterogeneity, time constraints, and group size. We primarily focused on evaluating the time to consensus in relatively small groups (up to a few tens of individuals) that are heterogeneous in individual preferences, certain traits (agreeability and persuasiveness), reputation, and the number and type of their closest social partners. Our models show that when allowing for individual differences there are significant time costs associated with shared decision-making. Here we discuss the main findings of our research and note some implications for theory and practice.

In our basic model, one individual (“listener”) interacts with another individual (“speaker”). Group members are motivated to reach a consensus and, thus, are willing to modify their opinions and/or preferences. As a result of an interaction, the listener shifts his preference in the direction of that of the speaker with the extent of the shift being proportional to the listener’s agreeability and the speaker’s persuasiveness. The listener is always chosen randomly from the group while the speaker can be chosen randomly, or with a probability proportional to their reputation, or from a subgroup of the listener’s closest friends. The time to consensus was defined as the time it takes for the absolute differences between individual opinions to drop to a certain proportion (e.g., 1/10th) of its initial value.

We found a simple approximation for the group’s final consensus value for several cases, and our results show that the largest influence on the consensus value is from the individuals with the largest value (*β*_*i*_/*α*_*i*_)*ρ*_*i*_ where *α*_*i*_, *β*_*i*_ and *ρ*_*i*_ are individual agreeability, persuasiveness, and reputation respectively. This implies that an individual who is, say, twice as persuasive, stubborn, and reputable as another will have eight times more influence on the consensus value.

With respect to the time to reach consensus, we have shown that decreasing the average persuasiveness or agreeability can significantly delay consensus. This is intuitive. Perhaps less intuitive is that the within-group variation in persuasiveness does not have much effect on the time to consensus, whereas within-group variation in agreeability can have a dramatic effect. One stubborn individual can delay the time to consensus and multiple stubborn individuals can have a significant impact on this time. Intuitively, if a group member is not persuasive enough to defend or promote their opinion, their opinion will be largely disregarded and the group will converge on the weighted average of the opinions of the other individuals. However, if one is not agreeable, then everybody else will converge on their opinion but this convergence can take a much longer time.

Time to consensus increases with the group size *n* but unless the group members vary highly in agreeability, the increase is relatively small. While larger groups need more interactions to reach a consensus, more interactions happen within the same time interval than in smaller groups. This reduces the overall effect of group size. However with large variation in agreeability, *τ* increases approximately linearly with *n*. This happens mostly because larger groups are more likely to have one or more highly stubborn individual. The effects of the variation in reputation *i* on time to reach consensus are relatively small but convoluted. In smaller groups, increasing the variation in reputations delays the consensus while in larger groups it speeds it up. These effects are relatively small though. Overall, the presence of a highly reputable individual (an “informal leader”) in the group is not enough to result in fast convergence of opinions and a rapid group action. The latter of course can be achieved if there is a formal leader (even if situational).

When we modify the model so that there are multiple, say ℓ, listeners per speaker the time to consensus decreases by a factor 1/ℓ. This result is intuitive as is a significant delay if individuals interact only with a subset of close social partners/friends. We have shown this numerically using three different types of networks common in the literature^45^ and represent contemporary organizational structures. For example, networks where everyone has the same number of connections can be found in business teams or military units, and groups with random connections are found in friendship networks and scientific collaborations^46^. We note that the scope of one-to-many communication has of course increased tremendously in the information age, and even more now with social media. This acts to reduce the time required for convergence of opinions. Social media of course also have an opposite effect by making multiple alternatives widely known.

Consensus can be achieved relatively quickly if there is a single individual who is stubborn, persuasive, reputable, and central to the social network. Such an individual would be an ideal candidate for a formal leader if decision speed is of primary concern. However the benefits of this speed may be outweighed by the costs group members incur due to the consensus value being strongly biased towards the value initially preferred by the leader. Our results (see Fig. 5 predict that fair leaders, i.e. who would be best in accounting for group members preferences, are highly, but not completely, stubborn. This is seen today where contemporary leaders tend to score low on agreeable personality scores^17^.

Our model is symmetric with regard to the direction of change of individual opinions. Ref. 47 studied a series of related models in which some individuals had a higher probability of switching from one of the two available options to another than in the opposite direction. Such a bias may result from the information available to individuals or some other reasons. Ref. 47 showed that a strongly biased minority can control the resulting consensus value, but the presence of unbiased individuals spontaneously inhibits this process, returning control to the numerical majority. Studying the effects of different biases on the time to convergence is an important direction for future work.

While a fast decision may be helpful, this is not the case if it comes at the expense of being wrong. Several animal studies have indicated the risks of collective decisions leading to sub- optimal outcomes through information cascades^48^,^49^ and speed/accuracy trade-offs^50^,^51^. This has implications for the common finding that larger and more diverse groups take longer to reach consensus, as these groups are also known to make more accurate decisions. Indeed, it is well established that more diverse groups can make better decisions than more uniform ones, the “wisdom of crowds” effect^52^. Similarly, real world human groups sometimes face a trade-off between decision speed and decision accuracy. This is the fundamental problem behind the well-known group think phenomenon^53^ and it has been used to describe example of poor group decision-making under a highly directive stubborn leaders such as Napoleon’s ill-fated attempt to conquer Russia and Kennedy’s Bay of Pig invasion of Cuba. Indeed, two facilitating conditions for group think are (a) an external crisis (which requires urgency) and (b) a highly directive leader. These conditions can sometimes lead groups to make a wrong decision.

Small-scale societies in humans, such as hunter-gatherer societies, tend to be egalitarian and lack formal leadership positions^54^. Nevertheless in these societies leadership emerges naturally in an informal way to simplify solving various coordination and collective action problems as well as resolve conflicts 8,10,55,56. Research on small-scale societies shows that leadership correlates with age, gender, and physical qualities, verbal skills and religious knowledge, trustworthiness, generosity and fairness, and the ability to build extensive social networks. Our models explicitly captured only few of these individual characteristics.

Here we have studied the dynamics of consensus building on relatively short within-generational time scales while assuming the existence of certain within-group variation. Our work is complementary with a number of recent papers which study evolutionary emergence and implications of leadership (reviewed in ref. 10). For example, refs 57 and 24 study emergence of leadership and hierarchy as a way to solve collective action problems. In their models group members accept leaders as a way to increase their individual benefits (even when leaders take a bigger share of the total reward). Ref. 58 modeled the emergence of (genetically differentiated) leaders and followers in the population composed by groups facing a coordination problem. They predict the emergence of a small number of “stubborn” leaders while most of the population become completely “agreeable”; consensus is prevented if there is more than one leader per group. This is complementary to our approach in which the distribution of personality traits is already stable and consensus always happens eventually. In other recent evolutionary models, leaders emerge as main contributors^59^ or the only contributors^60^ in public goods games (e.g., in volunteer dilemmas) with between-group conflict.

Our models and results shed light on why leadership is particularly common in our species, as humans live in large heterogeneous groups (with much variation in personalities, motivations, information available, and preferences). Under such conditions the groups may benefit greatly from having a hierarchical structure in place in which one or few individuals, the leaders, make a decision on behalf of the entire group. This hierarchical structure and reputation building as a knowledgeable or fair leader could have been aided – or even made possible – by the evolution of language which allowed individuals to affect/control preferences of many individuals at the same time with a low cost.

Leadership has been shown to increase group performance^55^ and the size of social networks and this may have been the case for our ancestors, helping them better survive hostile environments as they migrated and populated the globe^44^. Understanding the emergence of leaders is also important for understanding contemporary organizations. Whether in war, economics, or everyday decision-making, the problems that leaders and leadership face today may not be so different than what they faced before. Our results may provide an avenue to understanding how leadership impacts group decision-making under various conditions and a possible mechanism for how they emerge within the group. Finally, organizational scientists can potentially use these results to explore leader emergence in heterogeneous groups in order to facilitate group decision-making for professional organizations, businesses, or militaries.

## Additional Information

**How to cite this article**: Gavrilets, S. *et al*. Convergence to consensus in heterogeneous groups and the emergence of informal leadership. *Sci. Rep*. **6**, 29704; doi: 10.1038/srep29704 (2016).

## Supplementary Material

Supplementary Information
