# Supplementary material for: Convergence to consensus in heterogeneous groups and the emergence of informal leadership
Source: Sci Rep. 2016 Jul 14;6:29704. doi: 10.1038/srep29704 (PMC4944200; doi:10.1038/srep29704)
Supplement: Supplementary Information [file srep29704-s1.pdf]

Supplementary Information

Convergence to consensus in heterogeneous groups and the emergence of informal leadership

Sergey Gavrilets[1,2,3], Jeremy Auerbach[2], and Mark van Vugt[4]

[1]Department of Ecology and Evolutionary Biology, University of Tennessee, Knoxville, TN 37996, USA
[2]Department of Mathematics, University of Tennessee, Knoxville, TN 37996, USA
[3]National Institute for Mathematical and Biological Synthesis, University of Tennessee, Knoxville, TN 37996, USA
[4]Department of Social and Organizational Psychology, VU University of Amsterdam, Amsterdam, Netherlands

Our basic model describes the changes in preferences x_i in a group of n individuals ($i = 1,\ldots,n$) as a result of a stochastic sequence of events. Each events consists of an interaction between two individuals one of which (the listener) changes their preference in the direction of that of the other individual (the speaker). Specifically, we postulate that after an interaction between individual listener i and speaker j, the preference of i becomes:

$$x_i' = x_i + \alpha_i \beta_j (x_j - x_i), \tag{S1}$$

where α_i measures the listener's agreeability and β_j the speaker's persuasiveness.

Let ψ_{ij} be a random variable equal to 1 if the event includes individual i as a listener and individual j as a speaker and let $\psi_{ij} = 0$ otherwise. Let Ψ be a stochastic square matrix with elements $\Psi_{ij} = \alpha_i \beta_j \psi_{ij}$. Let matrix $S = I + \Psi - \mathrm{diag}(\Psi J)$, where I is the identity matrix, J is the column vector of 1's, and $\mathrm{diag}(\Psi J)$ is the diagonal matrix with the elements of vector ΨJ. Note that S is a stochastic matrix.Then the dynamics of vector $x = (x_1,\ldots,x_n)^T$ can be described by a system of stochastic linear equations

$$x_{t+1} = S_t x_t, \tag{S2}$$

where t specifies the event number. Let $E(S)$ be the expectation of S. The largest eigenvalue of matrix $E(S)$ is equal to 1. If the eigenvalue with the second largest modulus λ_2 of matrix $E(S)$ is smaller than 1 in absolute value, then all elements of $E(x)$ converge almost surely to a consensus value

$$x^* = v^T x_0,$$

where v is the left eigenvector of matrix $E(S)$ corresponding to eigenvalue 1.[1]

Assume first that each possible pair of individuals has an equal probability $\frac{1}{n(n-1)}$ to be chosen as a listener-speaker pair. In this case

$$E(S) = I + \frac{1}{n(n-1)} \left(\alpha \beta^T - n\overline{\beta}\ \mathrm{diag}(\alpha) \right), \tag{S3}$$

where α and β are vectors of α_i and β_i, respectively. Then the expectation of the consensus value is

$$x^* = \frac{\sum \frac{\beta_i}{\alpha_i} x_{i,0}}{\sum \frac{\beta_i}{\alpha_i}}. \tag{S4}$$

Next assume that at each event, the listener is chosen with equal probability out of all group members while the speaker is chosen out of remaining $n-1$ group members with probability proportional to their status ρ_i ($\sum \rho_i = 1$). In this case $\psi_{ij} = 1$ with probability $\frac{1}{n} \frac{\rho_j}{\sum_{j \neq i} \rho_j} = \frac{1}{n} \frac{\rho_j}{1-\rho_i}$. Now matrix $E(S)$ is given by equation (S3) with α_i substituted by $\alpha_i/(1-\rho_i)$ and β_i substituted by $\beta_i \rho_i$. Correspondingly, the expectation of the consensus value becomes

$$x^* = \frac{\sum \frac{\beta_i}{\alpha_i} \rho_i (1-\rho_i) x_{i,0}}{\sum \frac{\beta_i}{\alpha_i} \rho_i (1-\rho_i)}. \tag{S5}$$

Assume that interactions happen on a connected social network specified by an adjacency matrix C with element $c_{ij} = 1$ if a pair with listener i and speaker j is feasible. Assume that a listener is chosen randomly with a uniform probability while

the speaker is chosen randomly from $d_i = \sum_j c_{ij}$ potential speakers (e.g., neighbors or friends). In this case, for each feasible pair $\psi_{ij} = 1$ with probability $\frac{1}{nd_i}$. Then one can find $E(S)$ and x^* for any given adjacency matrix C.

In general finding or approximating the rate of convergence to τ is nontrivial (e.g.[2] and[3]). Assume that there are only $n = 2$ individuals i and j and that each individual has an equal chance to be a speaker or listener. Let $d = x_i - x_j$. Then $d_{t+1} = \xi_t d_t$, where ξ_t takes values $1 - \alpha_i\beta_j$ and $1 - \alpha_j\beta_i$ with equal probabilities. In this case, the asymptotic rate of convergence towards consensus is

$$\lambda = \frac{1}{2}\left(\ln(1-\alpha_i\beta_j) + \ln(1-\alpha_j\beta_i)\right).$$

This is based on the fact that as $t \to \infty$ the product $\prod_{i=1}^{t} \xi_i$ will have roughly $t/2$ terms $1 - \alpha_i\beta_j$ and $t/2$ terms $1 - \alpha_j\beta_i$ or, equivalently, t terms $\sqrt{(1-\alpha_i\beta_j)(1-\alpha_j\beta_i)}$.

Note that because $E(d_{t+1}) = E(\xi)^t d_0$, the rate of convergence can be approximated as

$$\lambda = \ln(E(\xi)) = \ln(1 - \frac{\alpha_i\beta_j + \alpha_j\beta_i}{2}).$$

The two equations above are close if $\alpha\beta \ll 1$.

With arbitrary n, perhaps the simplest approximation is to use the second largest eigenvalue λ_2 of matrix $E(S)$. This corresponds to approximating the dynamics of the stochastic system $x_{t+1} = S_t x_t$ with the deterministic system $x_{t+1} = E(S_t)x_t$. [From the above, we know that there is a bias even with $n = 2$.]

Assume that individuals have equal agreeability $\alpha_i = \alpha$ but differ in persuasiveness β_i. In this case,

$$E(S) = I + \frac{1}{n(n-1)}\alpha \begin{pmatrix} \beta_1 - n\overline{\beta} & \beta_2 & \dots & \beta_n \\ \beta_1 & \beta_2 - n\overline{\beta} & \dots & \beta_n \\ \dots & \dots & \dots & \dots \\ \beta_1 & \beta_2 & \dots & \beta_n - n\overline{\beta} \end{pmatrix},$$

where $\overline{\beta}$ is the average of β_i. The eigenvalues of $E(S)$ are $\lambda = 1$ with multiplicity one and

$$\lambda = 1 - \frac{\alpha\overline{\beta}}{n-1}$$

with multiplicity $n-1$. Correspondingly, the characteristic time scale of convergence to the consensus value can be approximated as

$$\tau = \frac{1}{\ln(1 - \frac{\alpha\overline{\beta}}{n-1})}\frac{1}{n} \approx \frac{n-1}{n}\frac{1}{\alpha\overline{\beta}}. \quad \text{(S6)}$$

Note that the factor $1/n$ is present because our unit of time corresponds to n events. Figure (S2) shows that this approximation works reasonably well in providing a lower bound on τ.

If individuals also differ in agreeability, one has to find the eigenvalues of matrix (S3). The characteristic equation of this matrix is known (e.g.,[4]) which allows one to approximate λ_2. However we find that a simple approximation

$$\tau = \frac{1}{\ln(1 - \frac{\alpha_h\overline{\beta}}{n-1})}\frac{1}{n} \approx \frac{n-1}{n}\frac{1}{\alpha_h\overline{\beta}}. \quad \text{(S7)}$$

where a_h is the harmonic mean of α_i, does a pretty good job in providing a lower bound on τ (see Figure S2 and Figure S4).

If individuals differ only in their status ρ_i but not in agreeability or persuasiveness ($\alpha_i = \alpha, \beta_i = \beta$) our approximation based on λ_2 of $E(S)$ predicts $\lambda = 1 - \frac{\alpha\beta}{n-1}$ and thus does not capture the dependence of τ on the distribution of ρ_i. Nevertheless the approximation provides a reasonable lower boundary on τ (see Figure S6). Figures (S8) numerically explores the dependence of τ on several parameters in more detail.

If individuals differ with respect to agreeability α_i, persuasiveness β_i, and reputation ρ_i and speakers are chosen with a probability proportional to their reputation, then the time to convergence is

$$\tau \approx \frac{1}{\mu}\frac{1}{\left(\frac{\alpha}{1-\rho}\right)_h \overline{\beta\rho}\, n} \quad \text{(S8)}$$

where $\left(\frac{\alpha}{1-\rho}\right)_h$ is the harmonic mean of $\frac{\alpha_i}{1-\rho_i}$ values and $\overline{\beta\rho}$ is the average of $\beta_i\rho_i$ values over the group.

Multiple listeners

Assume that each speaker is listened to not by one but by ℓ listeners. Figure (S10) shows that with ℓ listeners ($\ell = 2,4,8$) the time to convergence reduces approximately by a factor of $1/\ell$.

Interactions on social networks

These can be incorporated by postulating that the probabilities ψ_{ij} of i choosing j depend on i:

$$\Delta x_i = \frac{1}{n}\sum_{j\neq i}\frac{\psi_{ij}}{\sum_{k\neq i}\psi_{ik}}\alpha_i\beta_j(x_j - x_i), \tag{S9}$$

where ψ_{ij} is the adjacency matrix specifying the social network.

For example, in a simple case of a star network when individual #1 is connected to all other individuals and there are no other connections,

$$\psi = \begin{pmatrix} 0 & \frac{1}{n(n-1)} & \dots & \frac{1}{n(n-1)} \\ 1/n & 0 & \dots & 0 \\ 1/n & 0 & \dots & 0 \\ \dots & \dots & \dots & \dots \\ 1/n & 0 & \dots & 0 \end{pmatrix}.$$

Assuming no variation in agreeability and persuasiveness, the characteristic time scale is $\tau \approx \frac{1}{\alpha\beta}$, which is slightly longer than that in the basic case of a fully connected network (eq. S6). In the case of a circular network where each individual is connected only to its left and right neighbors,

$$\psi = \begin{pmatrix} 0 & 1/(2n) & 0 & \dots & 0 & 1/(2n) \\ 1/(2n) & 0 & 1/(2n) & \dots & 0 & 0 \\ 0 & 1/(2n) & 0 & \dots & 0 & 0 \\ \dots & \dots & \dots & \dots & \dots & \dots \\ 1/(2n) & 0 & 0 & \dots & 1/(2n) & 0 \end{pmatrix}.$$

In this case, S is a circulant matrix matrix which allows one to find its eigenvalues analytically. For example, with $n = 4,6$ and 8, $\tau = 1/(\alpha\beta), 2/(\alpha\beta)$ and $(2+\sqrt{2})/(\alpha\beta)$, respectively.

In the case of the star network, the central individual has $n-1$ times larger impact on the consensus value than each peripheral group member. In the case of a circular network, individual impacts on the consensus value are naturally the same.

In more general cases, matrix S loses the nice shape but the system remains linear. Figures (S11, S12, S13) show the dependence of τ on parameters in different networks.

Figure (S14) is analogous to Figure (5) of the main text but explores a wider range of parameters.

References

1. Tahbaz-Salehi, A. & Jadbabaie, A. Necessary and sufficient condition for consensus over random networks. *IEEE Transactions on Automatic Control* **53**, 791–795 (2008).

2. Boyd, S., Ghosh, A., Prabhakar, B. & Shah, D. Randomized gossip algorithms. *IEEE Transactions on Information Theory* **52**, 2508–2530 (2006).

3. Bajović, D., Xavier, J., Moura, M. & Sinopoli, B. Exact rate for convergence in probability of averaging processes via generalized min-cut. *IEEE Transactions on Decision and Control* (2012).

4. Zhivotovsky, L. & Gavrilets, S. Quantitative variability and multilocus polymorphism under stabilizing selection. *Theoretical Population Biology* **42**, 254–283 (1992).

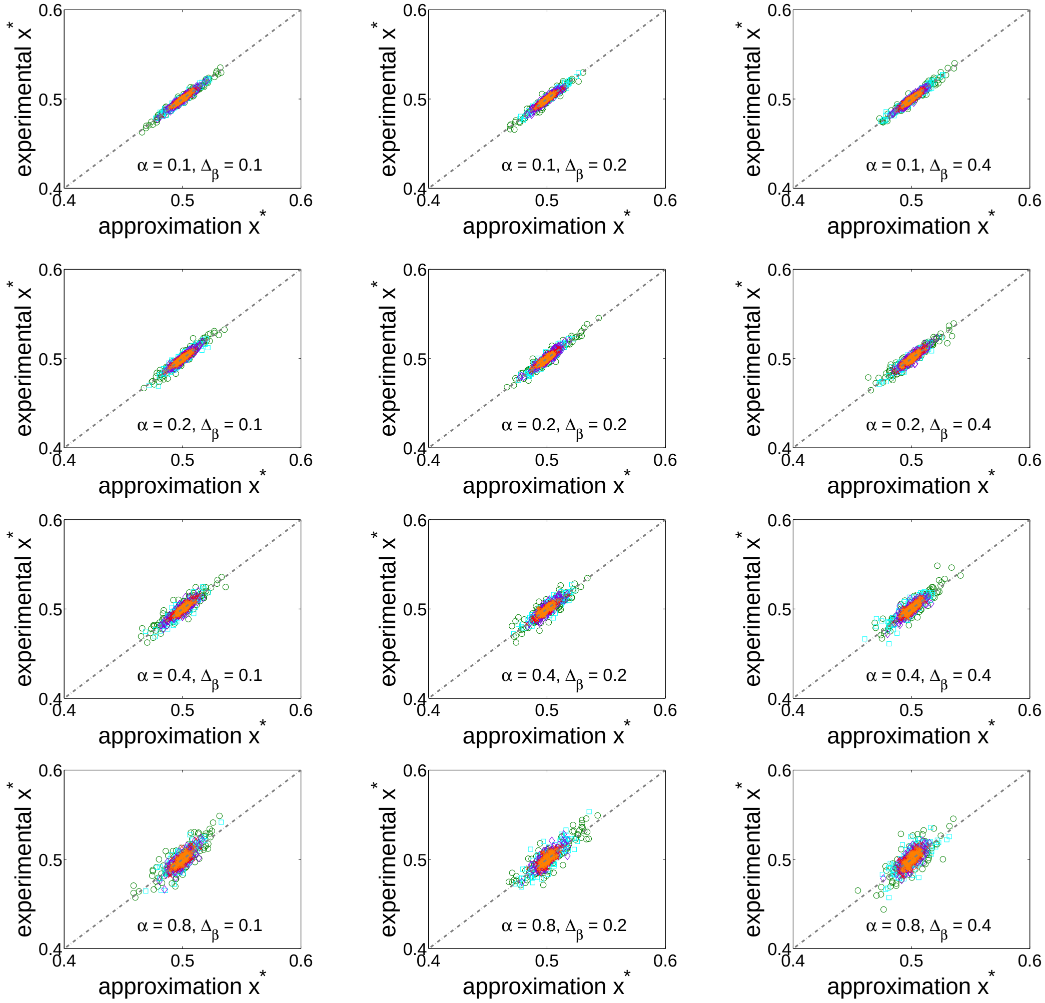

Figure S1. Comparison of approximation (S3b) with numerical results. Parameters α_i, β_i are chosen randomly and independently from a uniform distribution on the interval $0.5 \pm \Delta_\beta$. Initial preferences are drawn randomly from a uniform distribution on the interval (0,1) and all $\rho_i = 1/n$. The dashed line shows the diagonal. 200 parameter combinations; 200 runs for each parameter combination. Different group sizes correspond to different symbols: green circles ($n = 4$), blue squares ($n = 8$), purple diamonds ($n = 16$), red crosses ($n = 32$), orange asterisks ($n = 64$). $\mu = 1$.

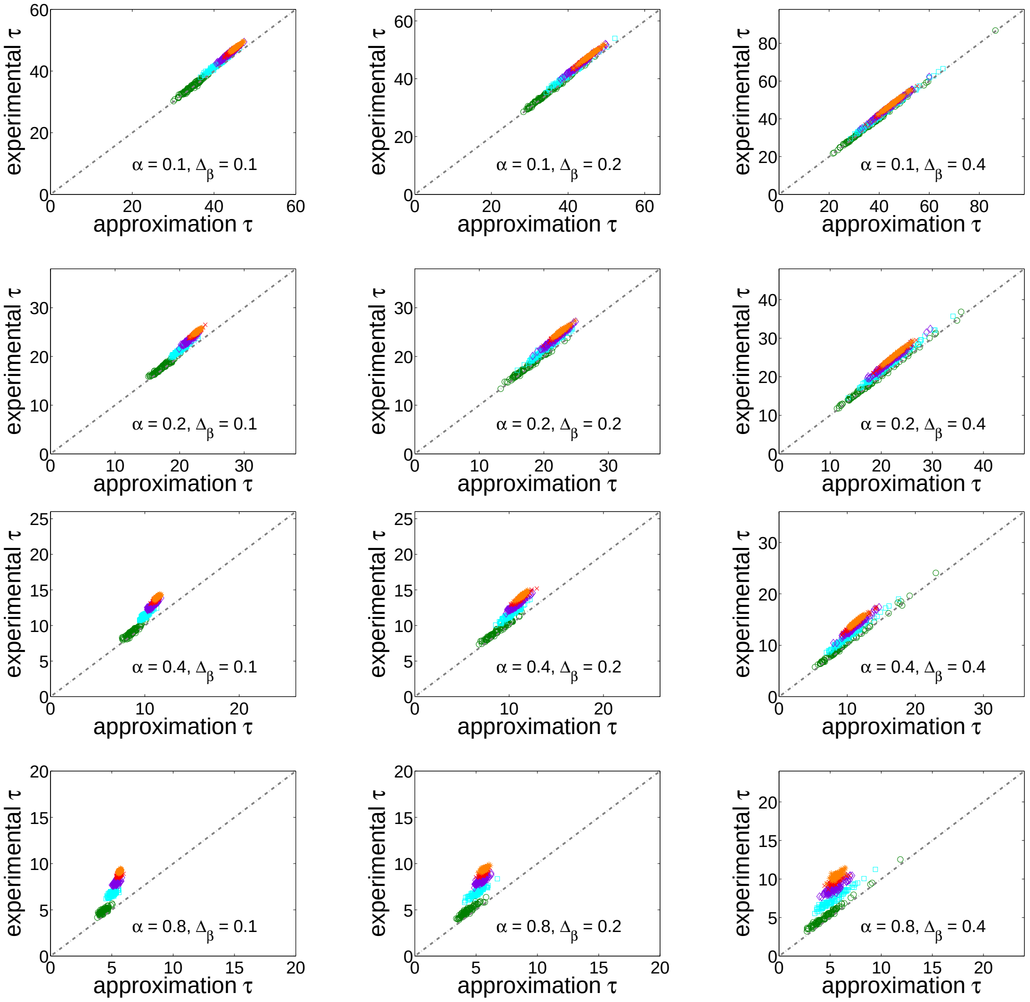

Figure S2. Comparison of approximation (S5) with numerical results. Parameters α_i, β_i are chosen randomly and independently from a uniform distribution on the interval $0.5 \pm \Delta_\beta$. Initial preferences are drawn randomly from a uniform distribution on the interval (0,1) and all $\rho_i = 1/n$. The dashed line shows the diagonal. 200 parameter combinations; 200 runs for each parameter combination. Different group sizes correspond to different symbols: green circles ($n = 4$), blue squares ($n = 8$), purple diamonds ($n = 16$), red crosses ($n = 32$), orange asterisks ($n = 64$). $\mu = 1$.

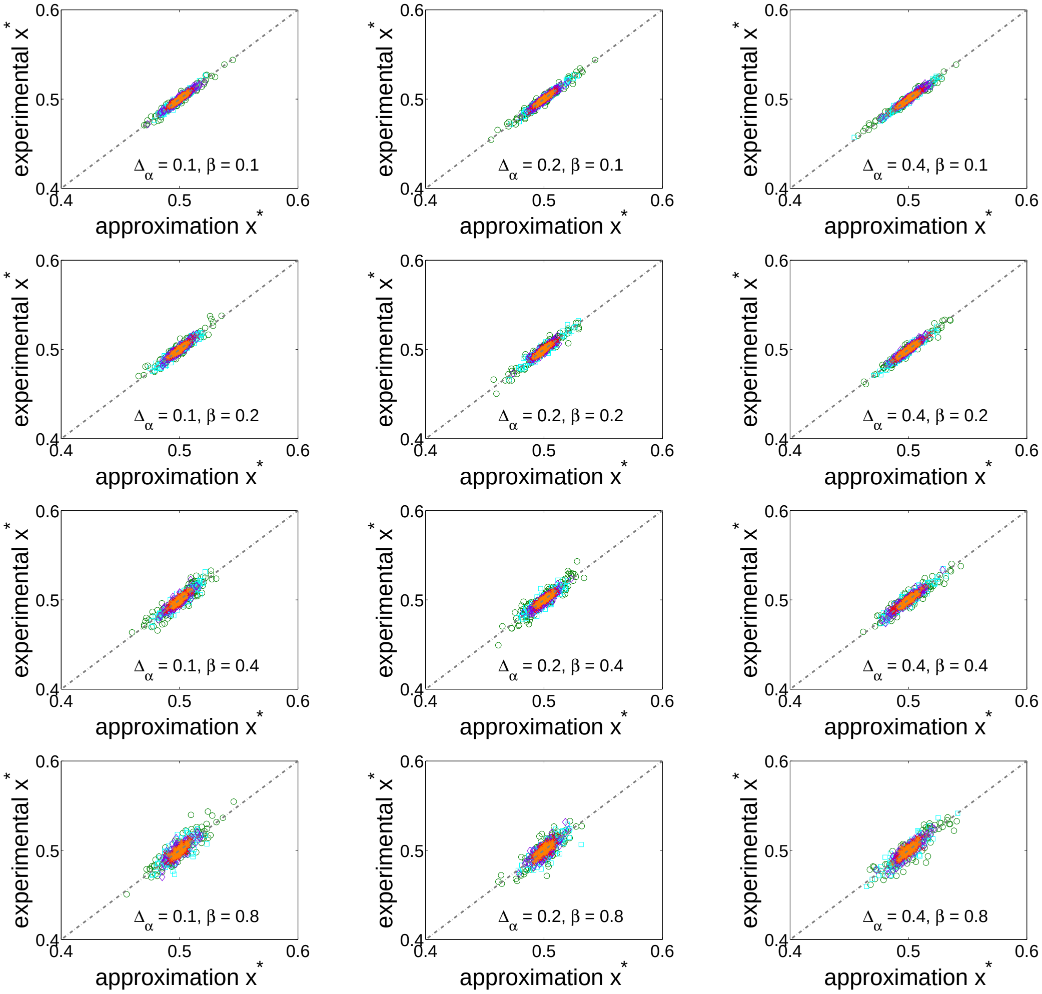

Figure S3. Comparison of approximation (S3b) with numerical results. Parameters α_i are chosen randomly and independently from a uniform distribution on $0.5 \pm \Delta_\alpha$. β_i are fixed at the values shown on the graphs. Initial preferences are drawn randomly from a uniform distribution on the interval (0,1) and all $\rho_i = 1/n$. The dashed line shows the diagonal. 200 parameter combinations; 200 runs for each parameter combination. Different group sizes correspond to different points: green circles ($n = 4$), blue squares ($n = 8$), purple diamonds ($n = 16$), red crosses ($n = 32$), orange asterisks ($n = 64$). $\mu = 1$.

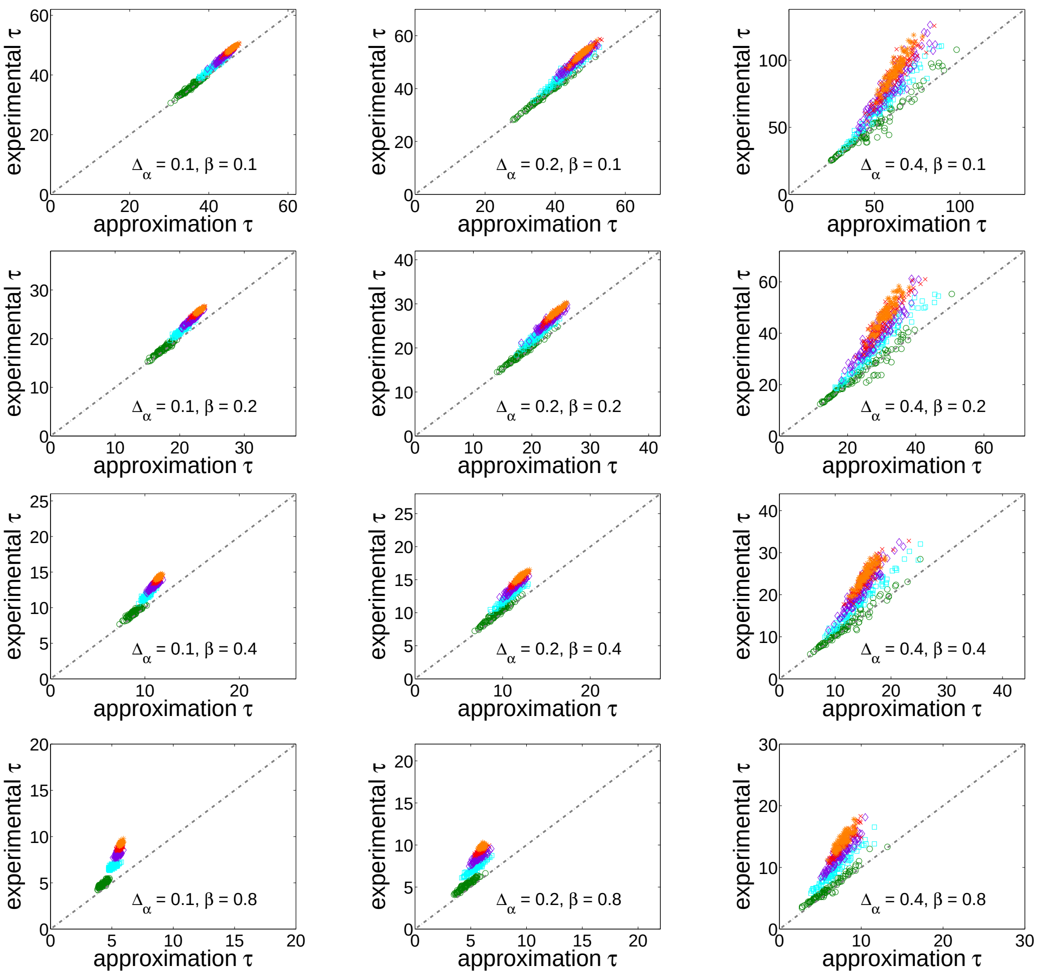

Figure S4. Comparison of approximation (S6) with numerical results. Parameters α_i are chosen randomly and independently from a uniform distribution on $0.5 \pm \Delta_\alpha$. β_i are fixed at the values shown on the graphs. Initial preferences are drawn randomly from a uniform distribution on the interval (0,1) and all $\rho_i = 1/n$. The dashed line shows the diagonal. 200 parameter combinations; 200 runs for each parameter combination. Different group sizes correspond to different symbols: green circles ($n = 4$), blue squares ($n = 8$), purple diamonds ($n = 16$), red crosses ($n = 32$), orange asterisks ($n = 64$). $\mu = 1$.

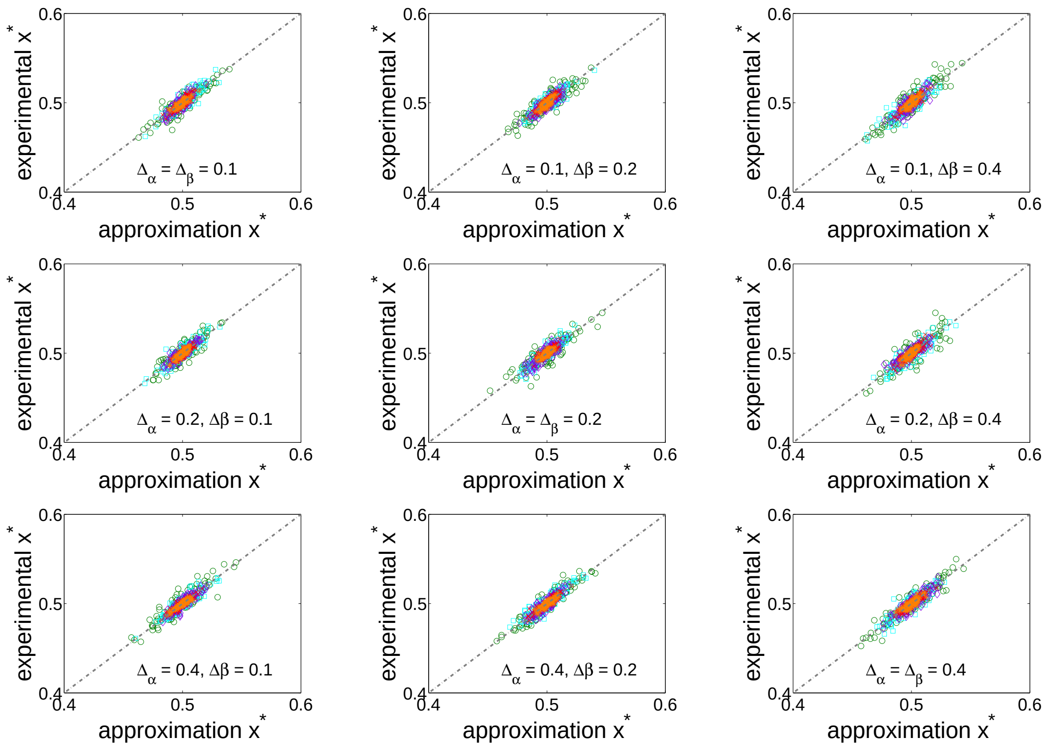

Figure S5. Comparison of approximation (S3b) with numerical results. All α_i and β_i are drawn from uniform distributions on $0.5 \pm \Delta_\alpha$ and $0.5 \pm \Delta_\beta$, respectively. Initial preferences are drawn randomly from a uniform distribution on the interval (0,1) and all $\rho_i = 1/n$. The dashed line shows the diagonal. 200 parameter combinations; 200 runs for each parameter combination. Different group sizes correspond to different symbols: green circles ($n = 4$), blue squares ($n = 8$), purple diamonds ($n = 16$), red crosses ($n = 32$), orange asterisks ($n = 64$). $\mu = 1$.

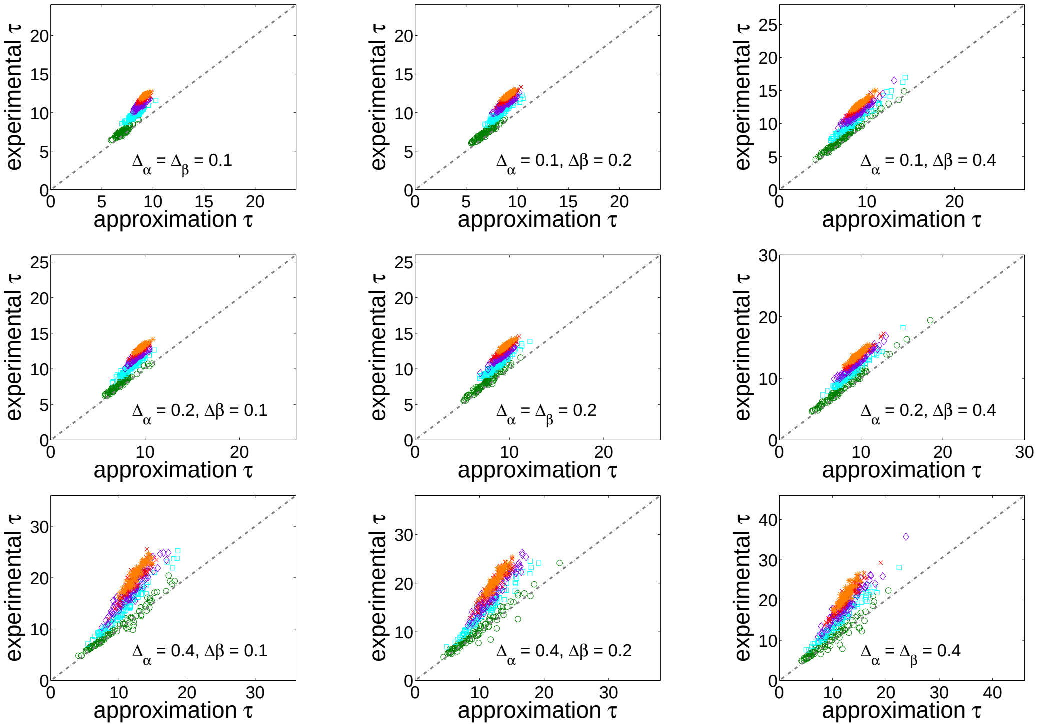

Figure S6. Comparison of approximation (S6) with numerical results. All α_i and β_i are drawn from uniform distributions on $0.5 \pm \Delta_\alpha$ and $0.5 \pm \Delta_\beta$, respectively. Initial preferences are drawn randomly from a uniform distribution on the interval (0,1) and all $\rho_i = 1/n$. The dashed line shows the diagonal. 200 parameter combinations; 200 runs for each parameter combination. Different group sizes correspond to different symbols: green circles ($n = 4$), blue squares ($n = 8$), purple diamonds ($n = 16$), red crosses ($n = 32$), orange asterisks ($n = 64$). $\mu = 1$.

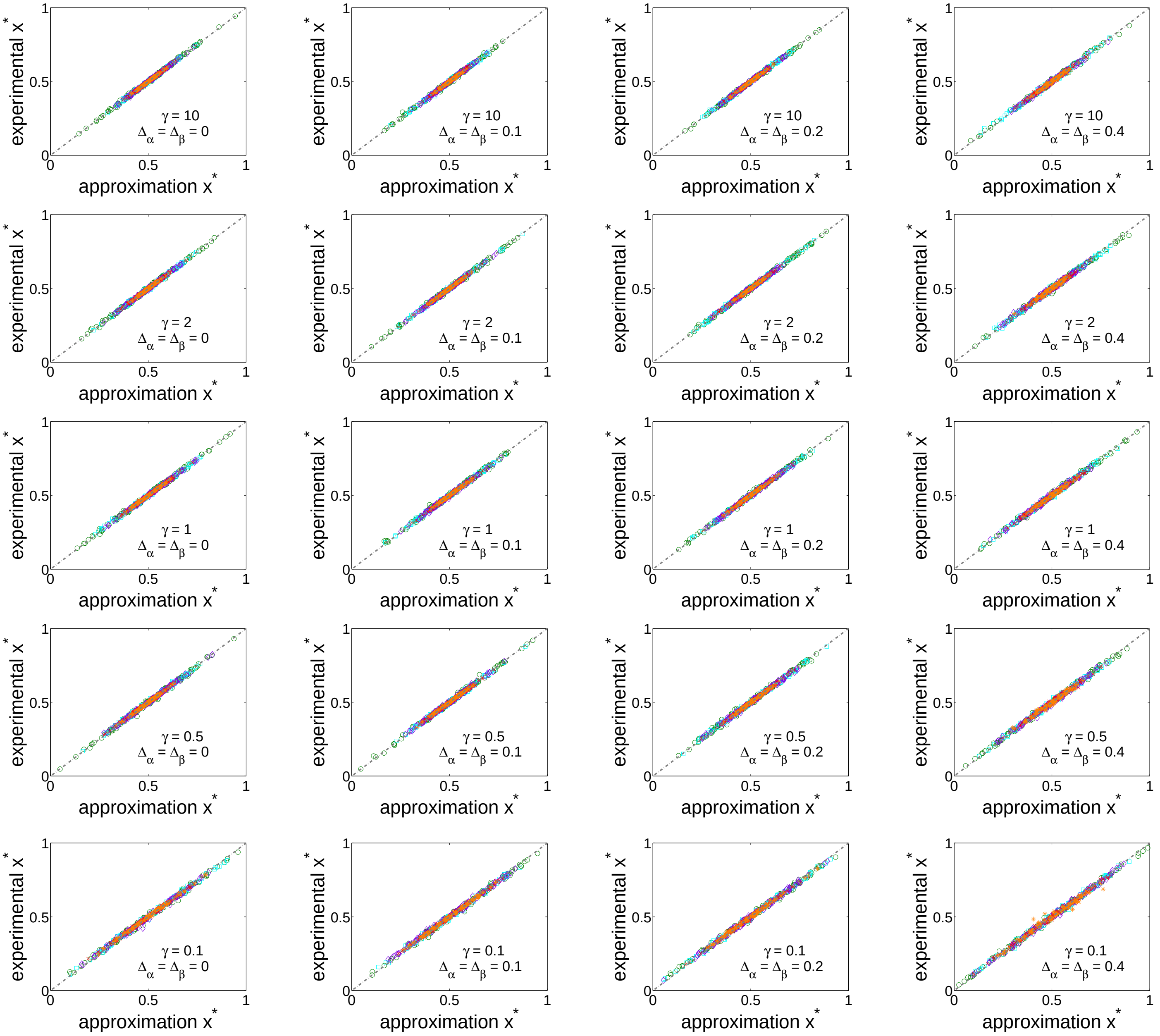

Figure S7. Comparison of approximation (S4) with numerical results. All α_i and β_i are drawn from uniform distributions on $0.5 \pm \Delta_\alpha$ and $0.5 \pm \Delta_\beta$, respectively. Initial preferences are drawn randomly from a uniform distribution on the interval (0,1) and reputation values ρ_i are chosen randomly from the symmetric Dirichlet distribution with different concentration parameters γ. When γ is small, most individuals have very low reputation while a few individuals will have a large reputation. The dashed line shows the diagonal. 200 parameter combinations; 200 runs for each parameter combination. Different group sizes correspond to different symbols: green circles ($n = 4$), blue squares ($n = 8$), purple diamonds ($n = 16$), red crosses ($n = 32$), orange asterisks ($n = 64$). $\mu = 1$.

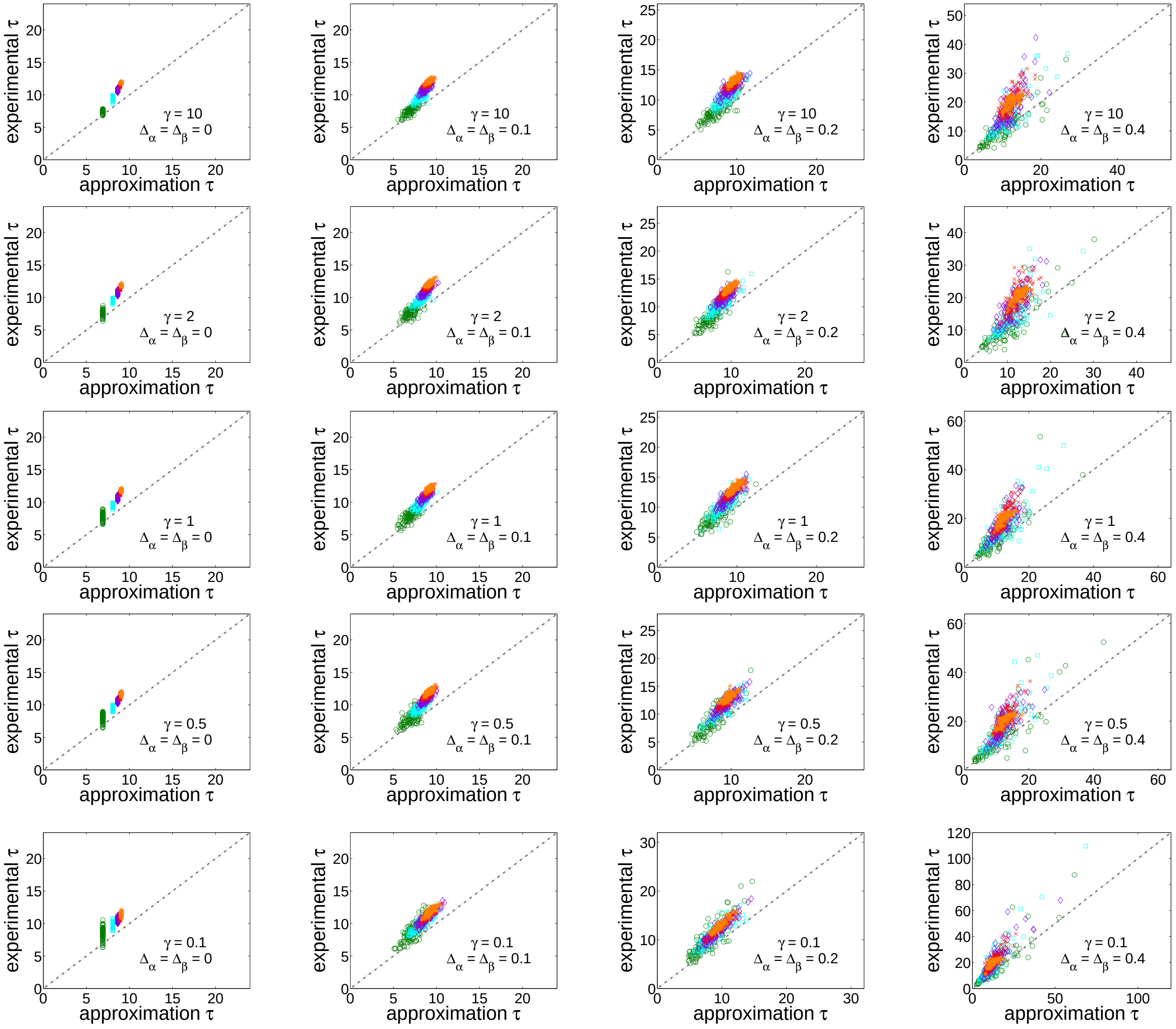

Figure S8. Comparison of approximation (S7) with numerical results. All α_i and β_i are drawn from uniform distributions on $0.5 \pm \Delta_\alpha$ and $0.5 \pm \Delta_\beta$, respectively. Initial preferences are drawn randomly from a uniform distribution on the interval (0,1) and reputation values ρ_i are chosen randomly from the symmetric Dirichlet distribution with different concentration parameters γ. When γ is small, most individuals have very low reputation while a few individuals will have a large reputation. The dashed line shows the diagonal. 200 parameter combinations; 200 runs for each parameter combination. Different group sizes correspond to different symbols: green circles ($n = 4$), blue squares ($n = 8$), purple diamonds ($n = 16$), red crosses ($n = 32$), orange asterisks ($n = 64$). Simulated results were scaled by n; $\mu = 1$.

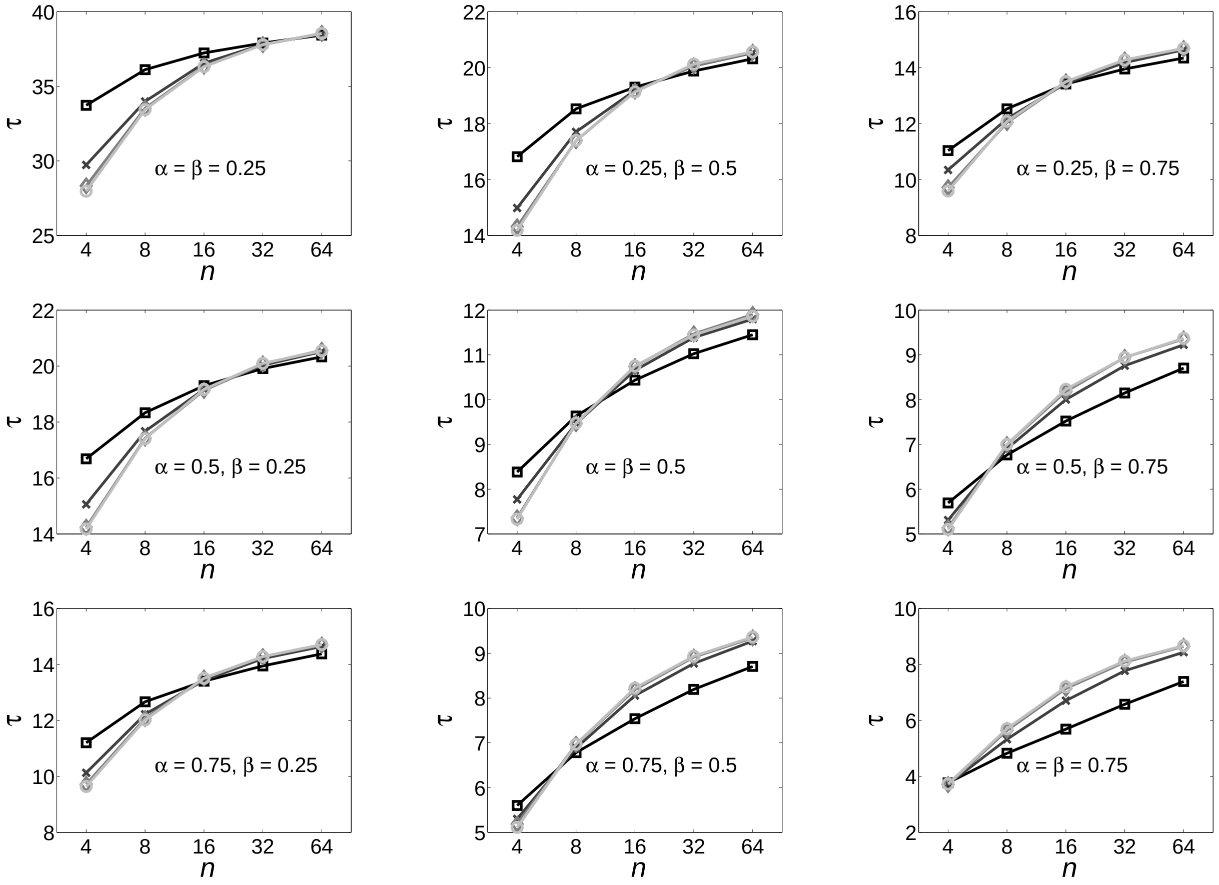

Figure S9. Characteristic time to consensus for groups with different distributions of reputation with no variation in personality traits. The lines with circles correspond to $\gamma = \infty$ (equal reputations $\rho_i = 1/n$), the lines with diamonds $\gamma = 10$ (uniform distribution of ρ), crosses $\gamma = 1$, and squares $\gamma = 0.1$ (a few highly reputable individuals). 10,000 simulations were run for each parameter combination and initial preferences were drawn from a uniform distribution on the interval (0,1).

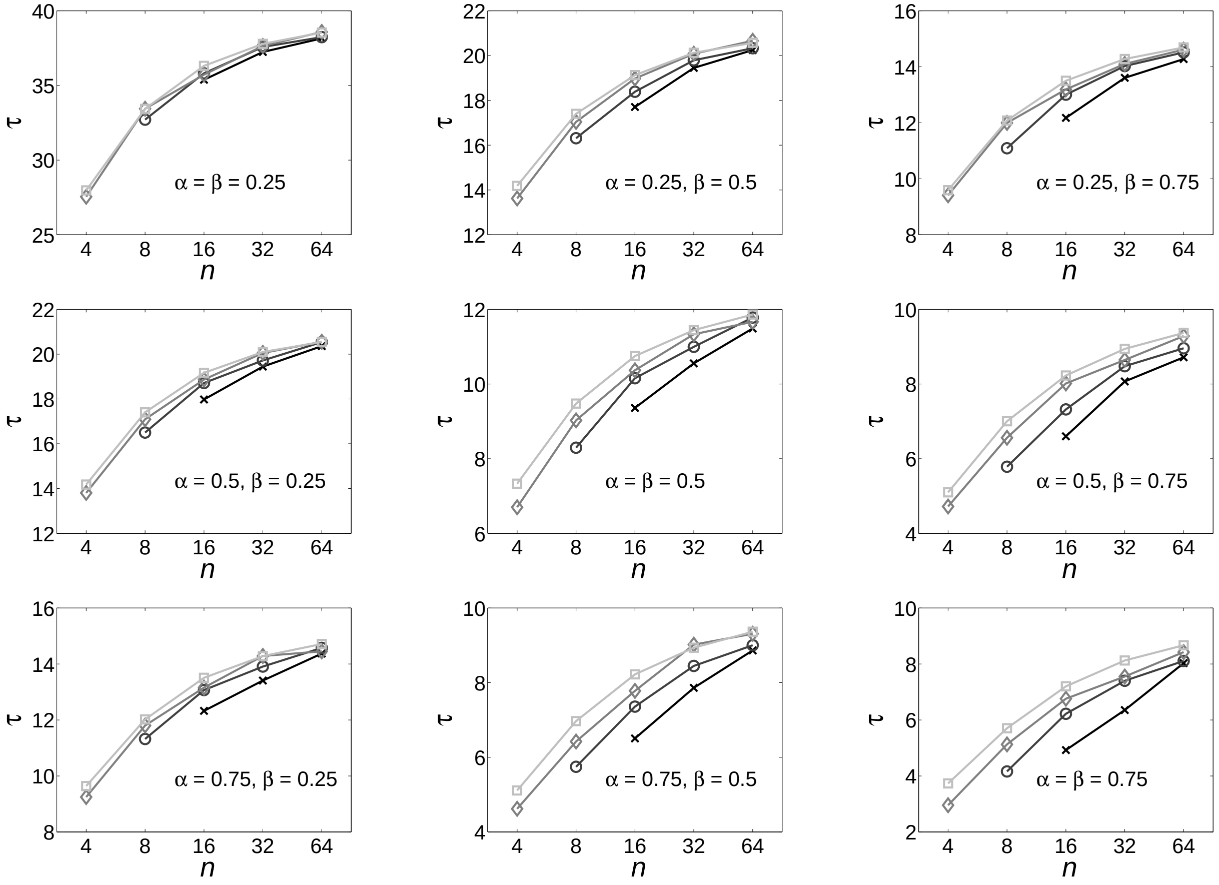

Figure S10. Characteristic time to consensus for groups with ℓ listeners chosen randomly and independently with no variation in personality traits. The lines with crosses correspond to $\ell = 8$, the lines with circles $\ell = 4$, diamonds $\ell = 2$, and the squares for one listener per interaction. Results were scaled by ℓ/n. 10,000 simulations were run for each parameter combination and initial preferences were drawn from a uniform distribution on the interval (0,1).

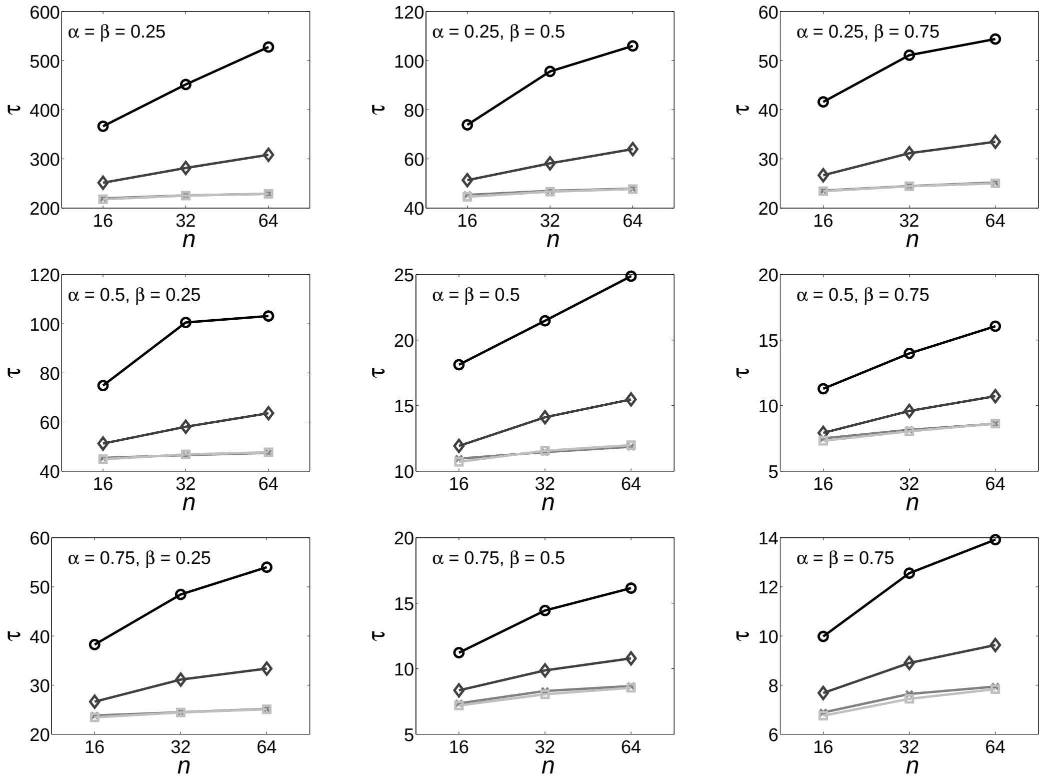

Figure S11. The average time to reach consensus for random networks where each group member has the same number of connections (k) and there was no variation in personality traits (α and β). The squares represent the average time to reach consensus for a complete network, the crosses denote networks with $n-1$ connections, the diamond networks had 8 connections per group member, and the circles had 4 connections per member. Reducing the number of connections increases the time to reach consensus. 5,000 simulations were run for each parameter combination with random formation of dyads and initial preferences were drawn from a uniform distribution on the interval (0,1).

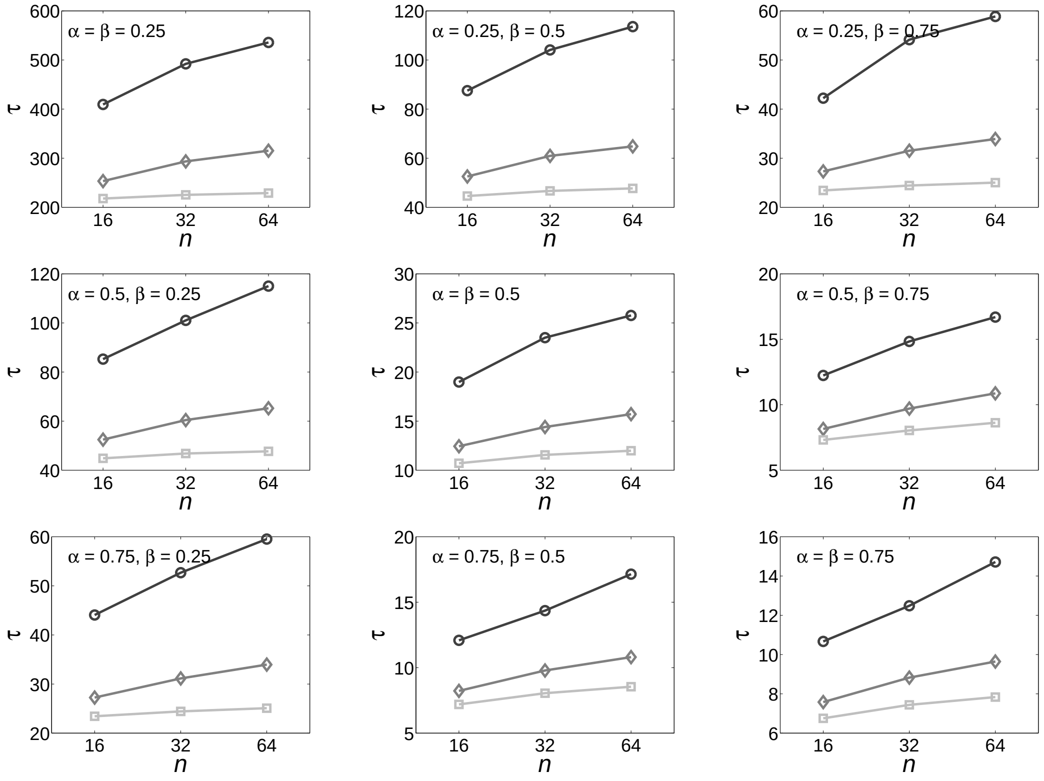

Figure S12. The average time to reach consensus for Erdös–Rényi networks with different probabilities of connectedness and no variation in personality traits (α and β). The squares represent the average time to reach consensus for a complete network, the diamonds denote networks with probability of connectedness of $8/n$, while the circles networks had a probability of $4/n$. A lower probability of connectedness increases the time to reach consensus dramatically but appears to have a reduced effect for larger group sizes. This could be due to networks having stubborn individuals with few connections, but larger groups having more interactions per time step mitigate this effect. 5,000 simulations were run for each parameter combination with random formation of dyads and initial preferences were drawn from a uniform distribution on the interval (0,1).

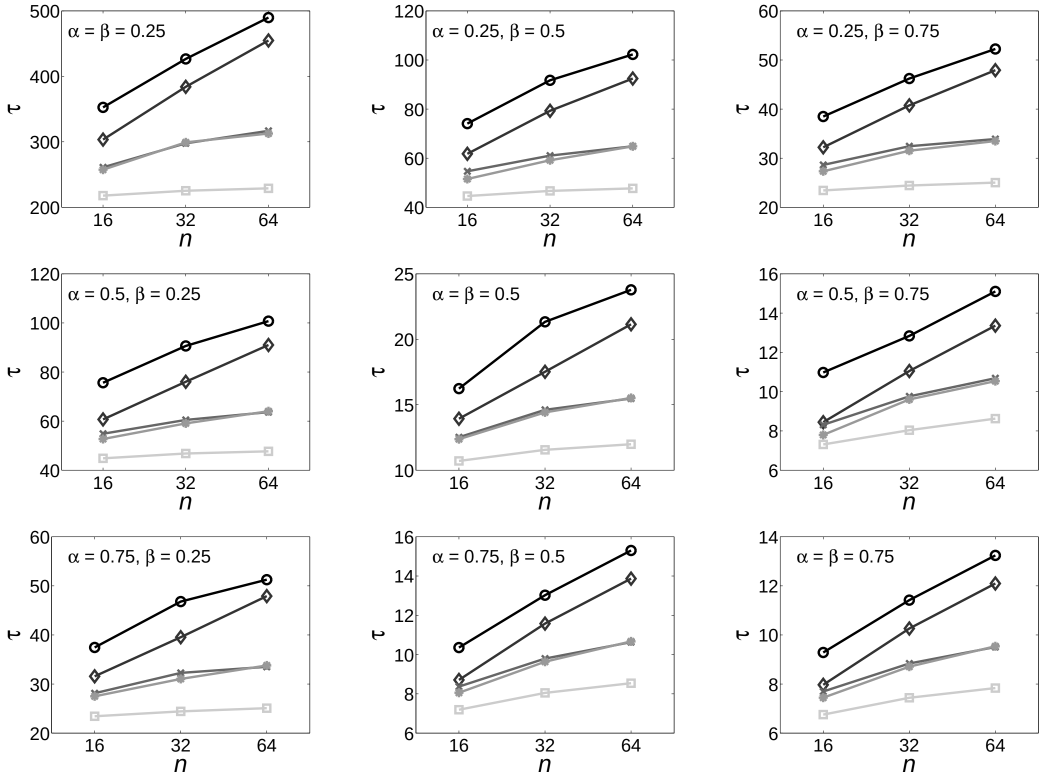

Figure S13. The average time to reach consensus for preferential attachment networks with no variation in personality traits (α and β), varying initial complete networks (m_0) and number of connections for each additional group members (m). The squares represent average time to converge for complete networks, the asterisks for the time when $m_0 = 8$ and $m = 4$, crosses for $m_0 = 8$ and $m = 2$, diamonds for $m_0 = 4$ and $m = 4$, and circles for $m_0 = 4$ and $m = 2$. Reducing the size of the initial complete network m_0 and therefore reducing the number of highly connected individuals dramatically increases the time to reach consensus similar to that found when reducing the number of connections with the other models. 5,000 simulations were run for each parameter combination with random formation of dyads and initial preferences were drawn from a uniform distribution on the interval (0,1).

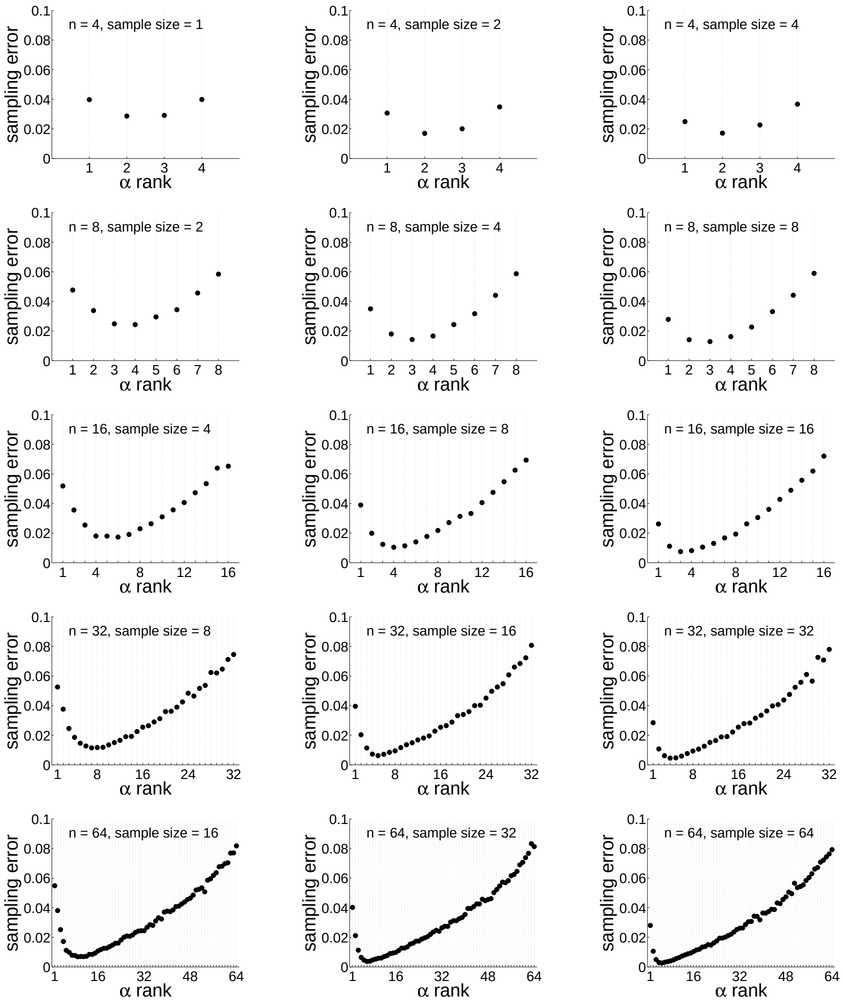

Figure S14. Average sampling errors, $(\tilde{x}_i - x_{opt})^2$, for individuals attempting to estimate the average initial preference $x_{\rm opt}$ by randomly sampling n' group mates in a group of size n. Individuals are ranked by their relative agreeability within the group from the lowest on the left to the highest on the right. Individual agreeability was chosen with a uniform probability from the interval (0,1) and persuasiveness was fixed at 1. 5,000 runs for each parameter combination with random formation of dyads and initial preferences drawn from a uniform distribution on the interval (0,1).
